# Irisin alleviated the reproductive endocrinal disorders of PCOS mice accompanied by changes in gut microbiota and metabolomic characteristics

**DOI:** 10.3389/fmicb.2024.1373077

**Published:** 2024-05-23

**Authors:** Meina Yang, Hongxia Deng, Siyu Zhou, Danhua Lu, Xiaoyang Shen, Lu Huang, Yan Chen, Liangzhi Xu

**Affiliations:** ^1^Reproductive Endocrinology and Regulation Laboratory, West China Second University Hospital, Sichuan University, Chengdu, China; ^2^Department of Obstetrics and Gynecology, West China Second University Hospital, Sichuan University, Chengdu, China; ^3^Key Laboratory of Birth Defects and Related Diseases of Women and Children (Sichuan University), Ministry of Education, Chengdu, China; ^4^Department of Public & Occupational Health, Amsterdam Public Health Research Institute, Amsterdam UMC, University of Amsterdam, Amsterdam, Netherlands

**Keywords:** irisin, polycystic ovary syndrome, folliculogenesis, gut microbiota, fecal metabolomics

## Abstract

**Introduction:**

Folliculogenesis and oligo/anovulation are common pathophysiological characteristics in polycystic ovary syndrome (PCOS) patients, and it is also accompanied by gut microbiota dysbiosis. It is known that physical activity has beneficial effects on improving metabolism and promoting ovulation and menstrual cycle disorder in PCOS patients, and it can also modulate the gastrointestinal microbiota in human beings. However, the mechanism remains vague. Irisin, a novel myokine, plays a positive role in the mediating effects of physical activity.

**Methods:**

Mice were randomly divided into the control group, PCOS group and PCOS+irisin group. PCOS model was induced by dehydroepiandrosterone (DHEA) and high-fat diet (HFD). The PCOS+irisin group was given irisin 400μg/kg intraperitoneal injection every other day for 21 days. The serum sex hormones were measured by radioimmunoassay. Hematoxylin and Eosin (H&E) Staining and immunohistochemistry (IHC) were conducted on ovarian tissue. The feces microbiota and metabolomic characteristics were collected by 16S rRNA gene sequencing and liquid chromatography-mass spectrometry (LC–MS).

**Results:**

In this study, we demonstrated that irisin supplementation alleviated reproductive endocrine disorders of PCOS mice, including estrous cycle disturbance, ovarian polycystic degeneration, and hyperandrogenemia. Irisin also improved the PCOS follicles dysplasia and ovulation disorders, while it had no significant effect on the quality of oocytes. Moreover, irisin could mitigate the decreased bacteria of Odoribacter and the increased bacteria of Eisenbergiella and Dubosiella in PCOS mice model. Moreover, irisin could alleviate the increased fecal metabolites: Methallenestril and PS (22:5(4Z,7Z,10Z,13Z,16Z)/ LTE4).

**Conclusion:**

These results suggest that irisin may alleviate the status of PCOS mice model by modulating androgen-induced gut microbiota dysbiosis and fecal metabolites. Hence, our study provided evidence that irisin may be considered as a promising strategy for the treatment of PCOS.

## 1 Introduction

Polycystic ovary syndrome (PCOS) is a reproductive endocrine and metabolic disease characterized by oligo-anovulation, hyperandrogenism, and polycystic ovarian morphology, affecting 4–21% of women of reproductive age worldwide (Lizneva et al., 2016). Metabolic disorders are characterized by insulin resistance, and hyperinsulinemia may be one of the key pathophysiological mechanisms and involved in the production of reproductive endocrine phenotypes such as excessive androgen synthesis and oligo-anovulation in PCOS (Wang et al., 2019). However, progress toward a cure for PCOS has been hindered by the absence of a clear understanding of the etiology (Witchel et al., 2019).

In recent years, due to the increased awareness of various risk factors, lifestyle interventions have been considered the first-grade and basic treatment for PCOS, including exercise interventions (Panidis et al., 2013; Liu et al., 2017). Studies show that physical exercises can not only relieve metabolic disorders but also have positive effects on decreasing androgen level, improving ovulation and menstruation, and finally increasing pregnancy rates in PCOS patients (Hakimi and Cameron, 2017; Shele et al., 2020; Dietz de Loos et al., 2021). However, because of the diversity of exercise types and the difficulty of achieving individualized exercise interventions, the most effective exercise prescription for managing PCOS is still unclear (Morris, 2021). Therefore, studying the molecular sensors of physical exercises and using them instead of exercises may be a more promising approach for disease treatment.

Irisin, a secreted peptide that is cleaved from cell membrane-associated fibronectin type III domain-containing protein 5 (Fndc5) (Boström et al., 2012), is a recently discovered exercise senor with a variety of biological functions, especially benefiting from metabolisms of glucose and lipids and maintaining the homeostasis of body energy (Perakakis et al., 2017; Liu et al., 2022; Waseem et al., 2022). As one of the reproductive endocrinal and metabolism disorders, some clinical studies have found changes in serum irisin levels in PCOS patients compared with healthy women, but the results were controversial (Bacopoulou et al., 2018; Wang et al., 2018; Chang et al., 2019; Luo et al., 2021; Onat et al., 2022). A recent experimental study found that irisin reduced the abnormal reproductive and metabolic phenotypes of PCOS in mice (Zheng et al., 2022), indicating that irisin may be involved in exercise relieving of PCOS phenotypes, but more studies are needed to confirm this hypothesis.

In recent years, it has been suggested that the gut microbiota is crucial for the onset, progression, and consequences of many chronic illnesses. Influencing the composition of gut microbiota and then alleviating chronic inflammation is an important mechanism for exercise to improve body health (Mach and Fuster-Botella, 2017; Motiani et al., 2020; Aragón-Vela et al., 2021). Several recent studies have reported that PCOS patients always bear kinds of gut microbe dysbiosis, and the regulation of microbiome and fecal metabolites could orchestrate PCOS phenotypes, suggesting that the gut microbiota may be a target for PCOS therapy (Qi et al., 2019; Han et al., 2021; Hussain et al., 2021). Nevertheless, it is unknown if irisin, the exercise sensor, may alter the gut microbiota and fecal metabolites to improve the PCOS phenotype. In this study, we found that irisin improved metabolism and reproductive endocrine disorders in PCOS mice; meanwhile, irisin also changed the gut microbiota and intestinal metabolite composition of PCOS mice, implying that irisin may participate in mediating the therapeutic effects of exercise on PCOS by affecting the gut microbiota and its metabolic pathways.

## 2 Materials and methods

### 2.1 Reagents

Antibodies against Cyp19a1, CX37/GJA4, GDF-9, LHR, and FSHR were obtained from Abcam (Cambridge, MA, United States). Antibodies against ERK, p-ERK, P38, p-P38, Cleaved-caspase 3, and Cyp19a1were purchased from Cell Signaling Technology (Waltham, MA, United States). DMEM/F12 medium, fetal bovine serum (FBS), penicillin, and streptomycin were purchased from Gibco (Grand Island, NY, United States).

### 2.2 Expression and characterization of purified recombinant irisin

Recombinant mouse irisin (r-irisin) was prepared following our previously published study (Qiao et al., 2016; Ma et al., 2018). In brief, the full-length coding sequence of Fndc5 was amplified from mouse cDNA and subcloned into a pTasy vector. Then, the irisin sequence was amplified from the plasmid using primers containing NdeI and XhoI site sequences. Then, the recombinant irisin plasmid was constructed by ligation with pET28a (+) vector at the NdeI and XhoI sites. After sequencing, to verify the correctness of the cloned irisin cDNA, the plasmids were transferred to *E. coli* strain Rosetta (DE3). The expression of r-irisin in *E. coli* was induced, purified by a nickel column, and diluted with PBS (pH7.4).Then, the concentration of the protein was determined. Overall, 30 nM r-irisin upregulated the phosphorylated p38 (p-p38) and phosphorylated ERK1/2 (p-ERK1/2) in 3 T3-L1 cells within 20 min, according to the methods described by R&D company (Qiao et al., 2016).

### 2.3 Animals

In total, 21-day-old female C57BL/6 mice purchased from Beijing Vital River Laboratory Animal Technology Co., Ltd. (China) were housed with four to five animals per cage under a 12-h light/dark cycle and in controlled temperature with free access to water and a diet. After 7 days of acclimatization, female mice were randomly divided into three groups: In the vehicle group, animals were injected daily with corn oil subcutaneously along with a control diet. In the PCOS group, animals were subcutaneously injected daily with DHEA (D4000; Sigma–Aldrich; 6 mg per 100 g, dissolved in corn oil) subcutaneously and were fed with a high-fat diet (HFD). In the PCOS +irisin group, animals were injected daily with DHEA subcutaneously, with 400 μg/kg r-irisin intraperitoneally every other day, and were fed with HFD. Here, the control diet comprised 12% fat, 73% carbohydrates (0% sucrose), and 19% proteins while the HFD comprised 60% fat, 26% carbohydrates (17% sucrose), and 25% protein. The mice were all treated with the above methods for 25 days before being sacrificed for further experiments. During treatment, vaginal smears were taken from the 13th day at the start of the intervention to observe estrous cycles. This project has ethical approval granted by the Medical Ethics Committee of West China Second University Hospital, Sichuan University (approval No. WCSUH21-2019-089).

### 2.4 Vaginal smears and estrous cycle determination

After the onset of intervention, vaginal smears were taken daily from the 13th day for 10 days to observe the estrous cycle at 9:00 a.m. In brief, 20 μL of 0.9% sterile saline was injected into the vagina using a pipette and gently swabbed repeatedly 3–4 times and then aspirated the saline mixed with the vaginal secretion from the mouse vagina and smeared it evenly on a slide. Then, after air drying at room temperature, the slices were stained with Wright’s solution, and the estrous cycle was determined by microscopic analysis. To evaluate the estrous cycle, the following can be observed: the proestrus is made up of nucleated epithelial cells; the estrus is defined by the predominance of cornified squamous epithelial cells; the metestrus is marked by the appearance of white blood cells and a decrease in cornified squamous epithelial cells relative to the estrus; the diestrus is defined by the predominance of white blood cells.

### 2.5 Measurement of hormone levels

Mice were anesthetized with an intraoral injection of chloral hydrate solution in a dose of 350 mg/kg body weight. Then, the blood was collected from the orbital venous plexus, and the serum was collected by centrifuging the whole blood at 4°C in 1200 g for 15 min. The serum levels of testosterone (T), follicle stimulating hormone (FSH), and luteinizing hormone (LH) were measured by radioimmunoassay (RIA) (Xin Fan Biotechnology, Shanghai, China).

### 2.6 Intraperitoneal glucose tolerance test (IPGTT) and insulin tolerance test (ITT)

The mice were fasted for 12 h before the IPGTT experiment and were given oral glucose of 50% concentration by gavage (in dose of 2.0 mg/kg body weight). Then, blood glucose (BG) levels were measured in the tail tip blood by 0 min (defined as fasting blood glucose or FBG), 15 min, 30 min, 60 min, and 120 min after gavage using a portable blood glucose meter (Accu-Chek Performa, Roche Diagnostics). For ITT, the mice were fasted for 6 h before 80 IU/kg body weight insulin (Wanbang Biopharmaceuticals, Shanghai, China) was intraperitoneally injected into each mouse, and BG at each time point was measured via the same method as IPGTT.

### 2.7 Hematoxylin and eosin (H&E) staining and immunohistochemistry (IHC)

After the mice were killed by cervical dislocation, the ovaries of each mouse were dissected and fixed in 4% paraformaldehyde for 24 h. According to the standard histological procedures, the maximum section (5 μm thick) of ovarian tissue in each sample was stained with hematoxylin and eosin. The number of different stages of follicles (primordial, primary, antral, and cystic follicles) was counted. To evaluate the effects of irisin on the expression of follicular development and atresia-related proteins, immunohistochemistry was conducted using the above paraffin sections. After routine dewaxing and hydration process, we incubated the tissue sections with 3% H_2_O_2_ solution for 10 min to remove endogenous catalase and then blocked the sections with 5% goat serum in 0.01 M PBS. After blocking, tissue was incubated with the primary antibody, and we used horseradish peroxidase (HRP)-labeled secondary antibody for further incubation, and diaminobenzidine (DAB) reagent was used for colorization. Hematoxylin was used for counterstaining the nuclei. Finally, gradient alcohol-xylene was used for tissue dehydration and transparency before the sections were mounted with neutral resin. The results of H&E staining and IHC were observed under an ordinary light microscope (Olympus CX33). The primary antibodies used here were as follows: anti-cleaved-caspase3 (1:400, 9664, CST), anti-GDF-9(1:2000, ab254323, abcam), anti-CX37/GJA4 (0.5–1 μg/mL, ab254323, abcam), and anti-P450 aromatase (Cyp19a1) antibody (1:200, ab18995, abcam).

### 2.8 Fecal DNA extraction and 16S rRNA gene sequencing

In total, 400 mg of fecal pellets were collected from each mouse in cryogenic vials immediately after being discharged before sacrifice. The fecal samples were snap-frozen with liquid nitrogen and were stored at −80°C before analysis. Fecal DNA extraction and 16S rRNA gene sequencing were carried out by Biomarker Technologies Co., Ltd. (Beijing, China) using the Pacbio sequencing platform. In briefly, fecal microbial DNA was extracted from mouse fecal samples using the TGuide S96 Magnetic Soil/Stool DNA Kit (Tiangen Biotech Co., Ltd., Beijing, China), according to the manufacturer’s instructions. The quantity and purity of the extracted DNA were controlled using NanoDrop 2000 (Thermo Scientific, United States). The integrity and size of the isolated DNA were evaluated using agarose gel electrophoresis. The full length of bacteria 16 s RNA gene was PCR-amplified using barcoded primers 27F_(16S-F) 5’-AGRGTTTGATYNTGGCTCAG-3′ and 1492R_(16S-R) 5’-TASGGHTACCTTGTTASGACTT-3′. Then, the PCR products were purified, quantified, and homogenized for constructing the sequencing library. The library repair kit (SMRTbell Template Prep Kit provided by PacBioperform) was used for damage repair, end repair, and adaptor attachment on a PCR instrument. Then, AMpure PB magnetic beads were used to recycle the final library, and a Sequel II sequencer was used for sequencing. After full length 16S rRNA sequences were obtained, the raw sequences were merged and quality filtered, and duplicates were removed by FLASH, Trimmomatic, and UCHIME, respectively. The resulting sequences were then aligned against the Greengenes database of 16S rRNA gene sequences. Reads were then measured by quantitative insights into microbial ecology (QIIME) analysis. The analyses of gut microbiota including alpha diversity, beta diversity, and difference significance analysis among groups were conducted using PD whole-tree, principal co-ordinate analysis (PCoA), and ANOVA, respectively.

### 2.9 Fecal untargeted metabolomics assay and analysis

In total, 50 mg of feces for each sample were used for metabolite extraction and were subjected to liquid chromatography-mass spectrometry (LC–MS), which was also carried out on Waters Xevo G2-XS QT of high-resolution mass spectrometer with Waters Acquity UPLC HSS T3 column (1.8 μm 2.1 × 100 mm) by Biomarker Technologies Co., Ltd. (Beijing, China), following the ordinary experimental protocol. Ultra performance liquid chromatography (UPLC) software (Waters ACQUITY UPLC HSS T3 C18, United States) was used to quantify the metabolite concentrations. The LC–MS data were analyzed by a supervised orthogonal partial least-squares discriminant analysis (OPLS-DA) clustering method. Variable Importance in Projection (VIP) derived from the OPLS-DA analysis was used to screen a variable, and a criterion of VIP value>1, *p* ≤ 0.01, and FC>1 was marked as a discriminating metabolite in this study. Biological pathway analysis was performed based on LC–MS data using MetaboAnalyst 4.0. The impact value threshold calculated for pathway identification was set at 0.1. Other instruments used in this study are shown in Supplementary Table S1.

### 2.10 Statistical analysis

All analyses were performed using SPSS (IBM SPSS Statistics 25). For unordered categorical variables and ordinal categorical variables, we used the χ^2^ test and non-parametric test, respectively. For continuous variables, mean ± SD (standard deviation) was used, and for others, we used median ± quartile range. For the comparison among the three groups, a one-way analysis of variance (ANOVA) was conducted, followed by Tukey’s test. Pearson correlation analyses were used for normal distribution, or Spearman correlation analyses were conducted. The significance level was set at *p* < 0.05. Immunohistochemistry and immunohistochemistry Western Blot data were collected and analyzed by Image J.

## 3 Results

### 3.1 R-irisin treatment did not improve IR and glucose metabolism in PCOS mice

**Figure 1:**
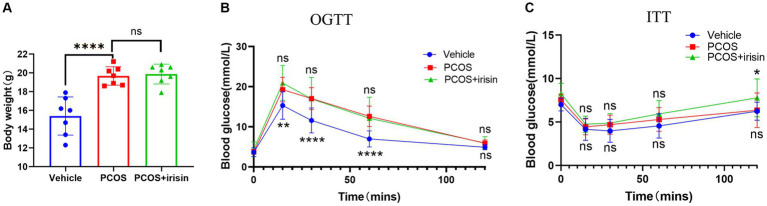
The effect of r-irisin on glucose metabolism in PCOS mice. **(A)** Bodyweight of each group of mice; **(B)** OGTT results of each group of mice; **(C)** ITT results of each group of mice; Sample size: *n* = 7 (7 mice from each group), Statistical method: ANOVA. Significance: *p<0.05, **p<0.01, and ****p<0.0001, ns means non-significance.

PCOS mice gained more weight than control mice, and the body weights of r-irisin-treated PCOS mice were not significantly different from PCOS mice (Figure 1A). To investigate the effect of irisin on glucose metabolism in PCOS mice, we performed the IPGTT and ITT trials. In the IPGTT test, we found that PCOS mice showed a significant increase in blood glucose (BG) after intraperitoneally given with glucose solution at each time point compared with control mice, but irisin treatment did not effectively improve the situation (Figure 1B). In the ITT test, we neither found that the BG of PCOS mice was significantly altered compared with the control mice after receiving insulin treatment, nor found a significant difference in the BG level in irisin-treated PCOS mice compared with PCOS mice. Only after 120 min of injecting insulin, higher BG in the PCOS + irisin group mice compared with mice in the PCOS group was found (Figure 1C). These results indicated that irisin did not improve IR and glucose metabolism in PCOS mice.

### 3.2 R-irisin treatment improved the reproductive endocrine disorders in PCOS mice

**Figure 2:**
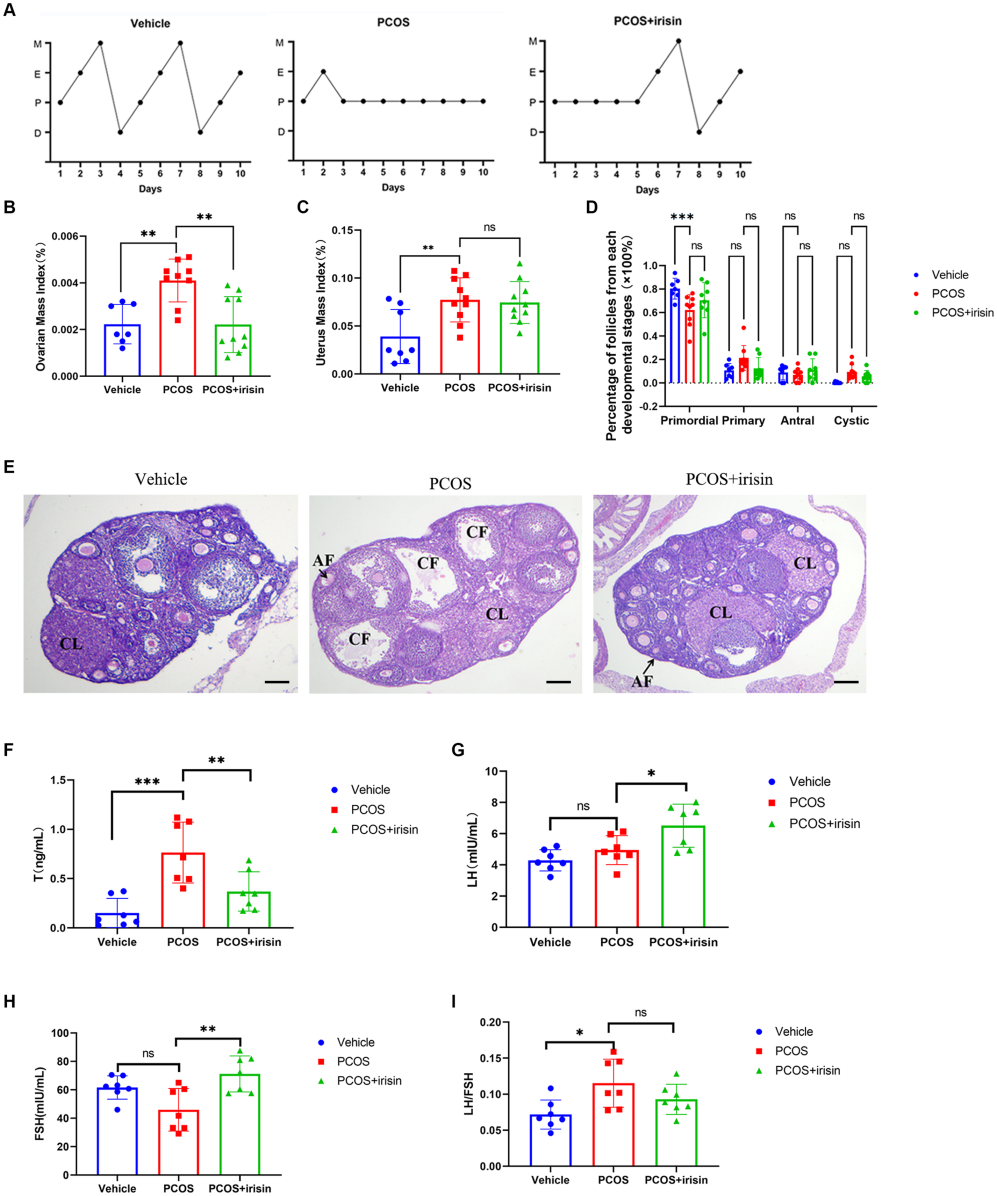
The effects of r-irisin on reproductive-endocrine function of PCOS mice. **(A)** Estrous cycles from each group of mice. M: Metestrus, E: Estrus, P: Proestrus, D: Diestrus; **(B)** Ovarian mass index (OI) of each group of mice. OI = ovary weight (g)/body weight; **(C)** The uterus mass index (UI) of each group of mice. UI = uterus weight (g)/body weight. **(D)** The proportion of the follicles at each developmental stage; **(E)** H&E staining of ovary sections from each group of mice. CL: corpus luteum, CF: cystic like follicle and AF: atretic follicle. The sale bar represents 200 μm. **(F)** The level of serum testosterone (T) from each group of mice; **(G)** the level of serum luteinizing hormone (LH) from each group of mice; **(H)** the level of serum follicle stimulating hormone (FSH) from each group of mice; **(I)** the LH/FSH ratio from each group of mice. Sample size: *n* = 7 (7 mice in each group); Statistical method: ANOVA. Significance: *p < 0.05, **p < 0.01, and ***p < 0.001, ns means non-significance.

Through observation of the estrous cycle of mice, we found that PCOS mice showed estrous cycle derangement, which showed near stagnation in the proestrus phase, whereas in control phase, each phase of the estrous cycle appeared more regularly. When treated with r-irisin, the appearance of each phase was partly restored in PCOS mice (Figure 2A). Similar to the phenotype of human PCOS, the ovary index (calculated by ovary weight/body weight of the same individual mouse) and uterus index (calculated by uterus weight/body weight of the same individual mouse) in PCOS mice increased significantly compared with control mice, while r-irisin treatment reduced the ovary index in PCOS mice, though the uterus index was not changed obviously (Figures 2B,C). Through H&E staining of ovary tissue sections, we found only a significant decrease in primordial follicles in PCOS mice ovaries. Although no statistical differences were presented, PCOS mice had an increased percentage of large cystic-like follicles compared with controls. In contrast, irisin treatment groups showed a reduction in cystic-like follicle number compared with PCOS mice. To be specific, the large cyst-like follicles which have very thinly and loosely arranged granulosa cells inside (commonly 2–3 layers) without oocyte can be regarded as specially atresia follicles and are considered as characteristic ovarian morphological changes in some PCOS mice models (Ryu et al., 2019) (Figures 2D,E).

In addition, by measuring serum steroid hormones using radioimmunoassay, we observed that PCOS mice had higher levels of testosterone (T), and a significant increase was also observed in LH/FSH ratio than control mice. However, there was no significant change in the level of LH and FSH between PCOS and the control groups. After being treated with r-irisin, the level of T went down, but the LH and FSH levels were unexpectedly increased, accompanied by a slight decrease in LH/FSH ratio without statistical differences when compared with PCOS mice (Figures 2F–I). These results indicated that exogenous irisin supplementation ameliorated the reproductive endocrine-related pathological phenotypes in PCOS mice.

### 3.3 R-irisin altered the expression of follicle development/atresia-related markers in PCOS ovaries

**Figure 3:**
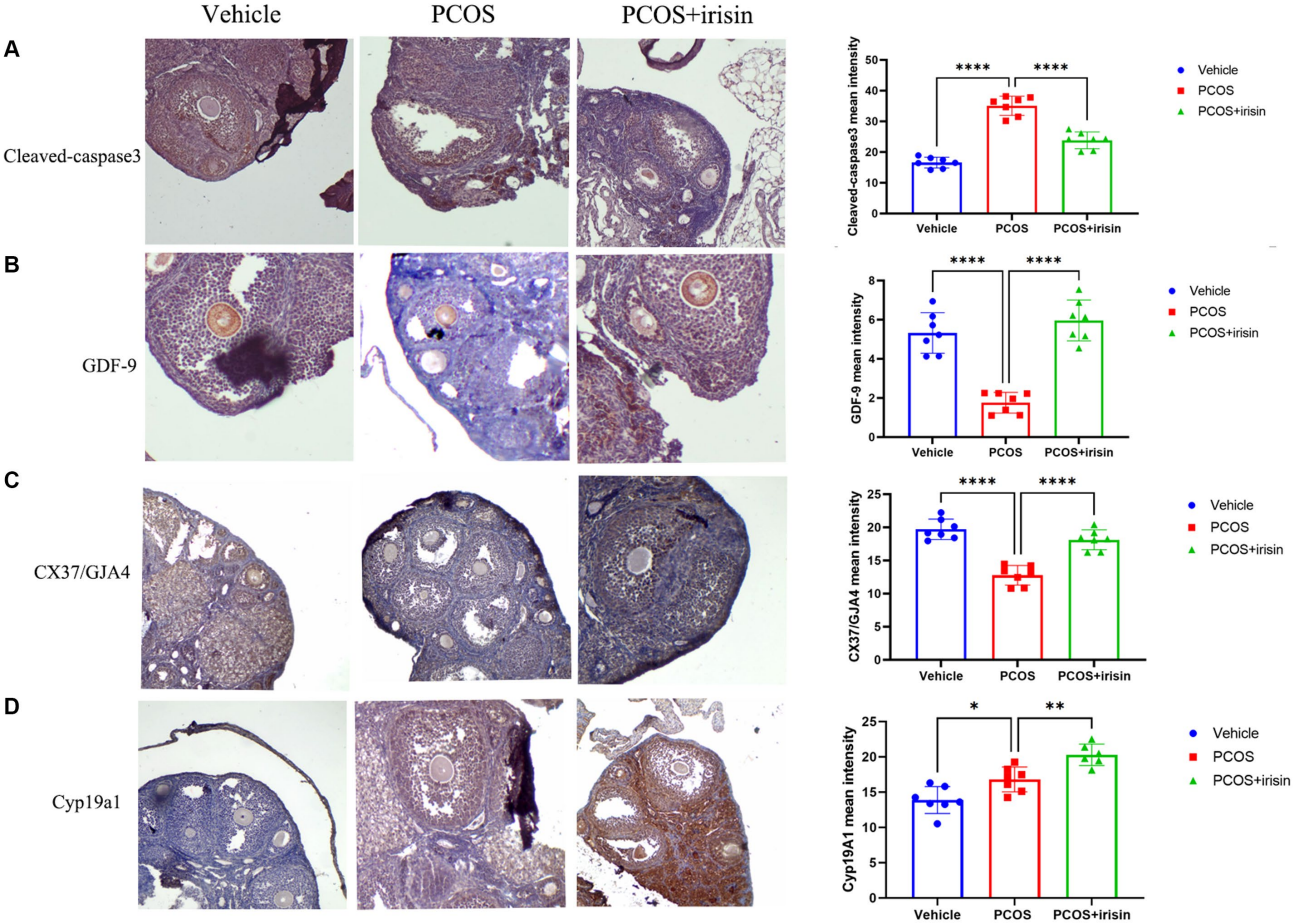
The effect of r-irisin on the expression of follicle development and atresia-related proteins in vivo. **(A)** Immunostaining of cleaved caspase-3 in ovary sections from each group of mice. **(B)** Immunostaining of GDF-9 in ovary sections from each group of mice. **(C)** Immunostaining of the granulosa cell-oocyte bridge protein CX37/GJA4 in ovary sections from each group of mice. **(D)** Immunostaining of CYP19a1 in ovary sections from each group of mice. The bar charts in the right column were the immunohistochemical semiquantitation depending on average light intensity. Sample size: *n* = 7 (7 mice in each group). Statistical method: ANOVA. Significance: *p < 0.05, **p < 0.01, and ****p < 0.0001.

To further explore the mechanism by which r-irisin altered the ovarian pathological phenotype of PCOS mice, we used immunohistochemistry to examine the expression of some proteins involved in follicle development or atresia. The results demonstrated that the expression of cleaved-caspase-3, the marker for follicle atresia, in the ovarian granulosa cells was significantly increased in PCOS mice compared with the control mice; however, the expression of GDF-9 in oocyte, a positive regulator of follicle development, and the expression of the bridge protein CX37/GJA4 in granulosa cells, which mediates the connection and communication between oocyte and granulosa cells, were significantly reduced in PCOS ovaries compared with the control. After r-irisin treatment, expression of cleaved-caspase-3 in PCOS ovary granulosa cells was decreased, and the expression of GDF-9 in oocytes and CX37/GJA 4 in granulosa cells was restored (Figures 3A–C). There was also a significant increase of cyp19a1 (aromatase) expression in r-irisin-treated mice ovaries (Figure 3D). These results suggested that r-irisin improving the ovarian pathological phenotype in PCOS mice may be related to its participation in the regulation of follicle development and atresia.

### 3.4 R-irisin altered gut microbial diversity of PCOS mice

**Figure 4:**
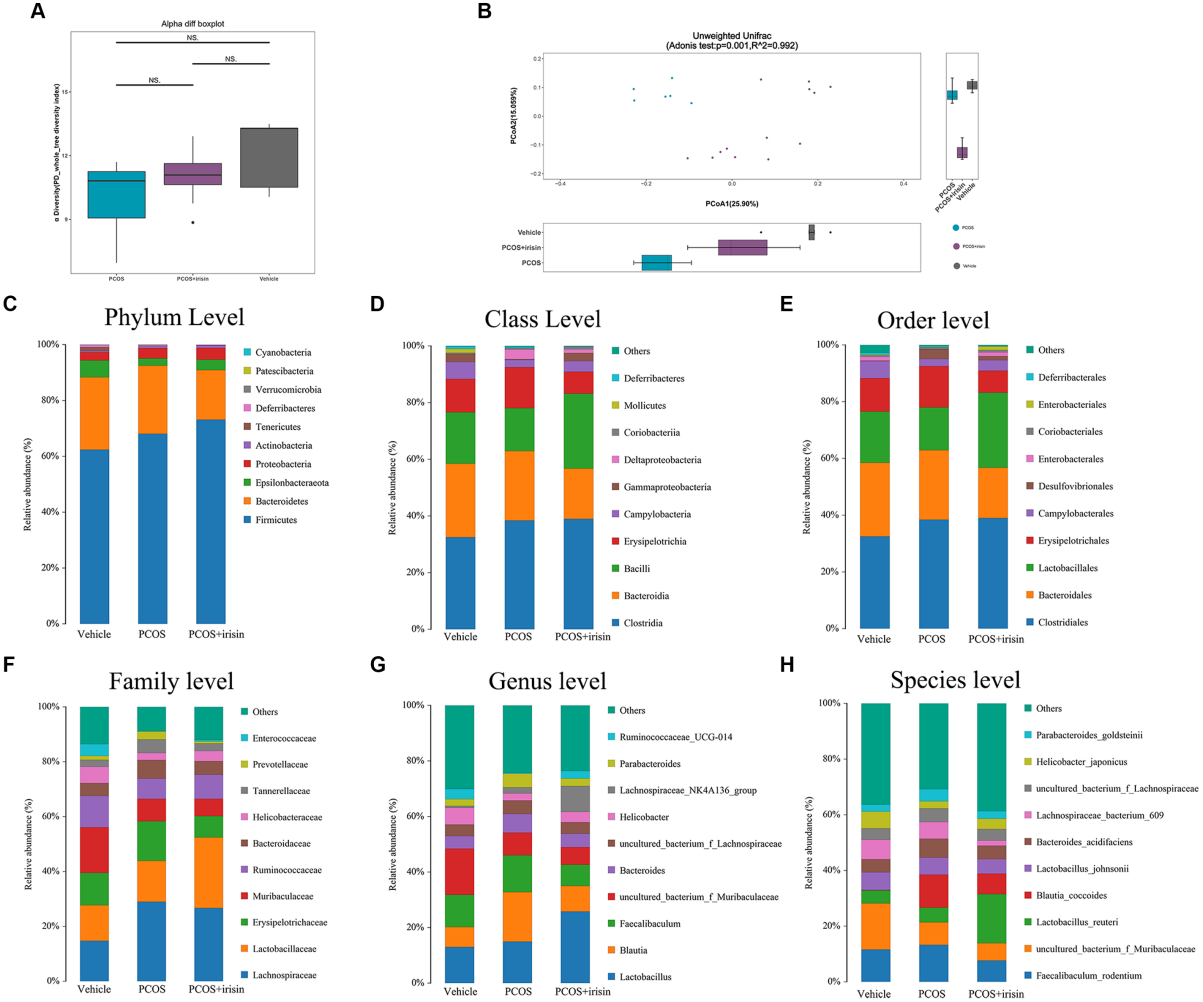
R-irisin treatment improved beta diversity of the gut microbiota in PCOS mice. **(A)** Alpha diversity of the gut microbiota from feces samples of each group of mice displayed by the phylogenic gut microbiota diversity whole tree index. **(B)** Beta diversity of the gut microbiota was performed using the PCoA analysis using unweighted UniFrac distance. Numbers on the axes represent the percentage of variation interpreted by that coordinate. **(C–H)** Gut microbial composition in each group of mice in phyla level **(C)**, class level **(D)**, order level **(E)**, family level **(F)**, genus level **(G)**, and species level **(H)**. Sample size: vehicle group (*n* = 5), PCOS group (*n* = 6) and PCOS +irisin group (*n* = 8). *p < 0.05, **p < 0.01, and ***p < 0.001.

By performing 16S RNA sequencing of mice feces, the gut microbiome data were acquired. Through principal component analysis (PCA), we found that there was no significant difference in alpha diversity in the gut microbiome (PD whole tree Index) (Figure 4A), whereas there were significant differences in beta diversity among the three groups (vehicle, PCOS, and PCOS + irisin groups). From the perspective of the beta diversity, principal coordinate analysis (PCoA) based on the unweighted UniFrac distance revealed that samples from three groups of mice formed distinct clusters significantly far apart (Figure 4B). We then analyzed and compared the composition of the gut microbiota of the three groups of mice. From the species relative abundance bar chart, we found that, at the phylum level, Firmicutes, Bacteroidetes, Epsilonbacteraeota, and Proteobacteria were in largest proportions in the three groups of mice; at the level of the class, Clostridia, Bacteroidia, Bacilli, and Erysipelotrichia were in largest proportions in the three groups; at the level of order, Clostridiales, Bacteroidales, Lactobacillales, and Erysipelotrichales accounted for the largest proportion in the three groups; at the level of family, Lactobacillaceae, Erysipelotrichaceae, and Muribaculaceae occupied the largest proportion in the three groups; at the genus level, *Lactobacillus*, *Blautia*, and *Fecalibaculum* accounted for the largest proportion in the three groups; at the level of species, *Fecalibaculum_rodentium*, *uncultured_bacterium_f_Muribaculaceae*, *Lactobacillus_reuteri*, and *Blautia_coccoides* accounted for the largest proportion in the three groups. We can also observe that the gut microbiota of the three groups of mice did not differ significantly in species composition characteristics, but the proportion of different species varied in the classification level of each species and became the basis for the significant differences in microbial beta diversity (Figures 4C–H).

### 3.5 R-irisin altered the abundance of gut germ strains at different species classification levels in PCOS mice

**Figure 5:**
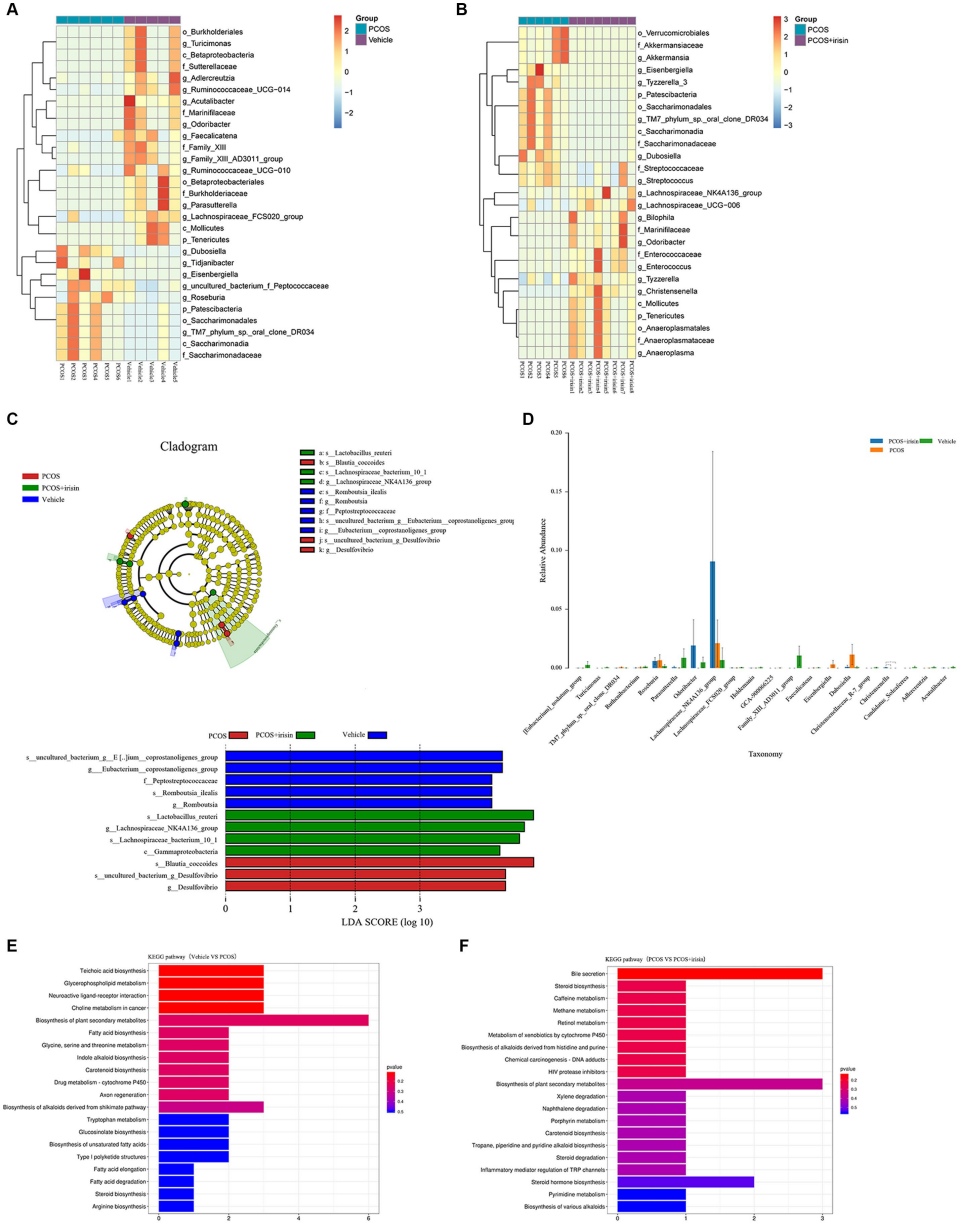
Profiling of the gut microbiota from each group of mice. **(A,B)** Wilcoxon heat maps showing differentially detected abundant strain taxa between PCOS and vehicle group **(A)**, and between PCOS +irisin and PCOS group **(B)**. These taxa were identified by the Wilcoxon test with *p* < 0.05. Cladogram and bar plot of the LEfSe analysis showing the most characteristic taxa in each group of mice (taxa with LDA score > 2.0). The variance analysis of abundant taxa at the genus level among three groups of mice. **(E,F)** Differentially key altered metabolic pathways between PCOS and vehicle group **(E)** and between PCOS +irisin and PCOS group **(F)** annotated by the KEGG analysis. Sample size: vehicle group: *n* = 5; PCOS group: *n* = 6 and PCOS +irisin group: *n* = 8. Significance: +, *p* < 0.10; *p < 0.05; **p < 0.01; and ***p < 0.001.

Using the Wilcoxon analysis, the heat maps of differential abundance between the vehicle versus PCOS and PCOS versus PCOS + irisin group were constructed, respectively, and further showed that DHEA and HFD exposure significantly changed the abundance of bacteria at the different species classification levels, while r-irisin treatment also changed the abundance of multiple strains in the gut flora of PCOS mice at the multispecies level (Figures 5A,B). As these differences are more common at the genus level, we extracted the strains with abundance differences at this level for ANOVA and found that r-irisin reversed the decrease of the abundance of *Odoribacter* and the increase of *Eisenbergiella* and *Dubosiellad* in PCOS mice caused by DHEA and HFD exposure at this level (Figure 5C). Furthermore, through LEfSe analysis, using LDA score > 2.0 as the screening criterion, we also found that the three groups of mice had their remarkable signature intestinal bacteria at the genus level with statistical differences (Figure 5D). Furthermore, Kyoto Encyclopedia of Genes and Genomes (KEGG) analysis was performed using the predicted gene abundance to understand the functional contribution of gut microbial ecology. The results showed that r-irisin treatment might involve in the regulation of genes related to bile acid secretion, plant secondary metabolite biosynthesis, hormone stimulation, hormone biosynthesis, and ovarian steroid biosynthesis pathways (Figures 5E,F).

### 3.6 DHEA plus HFD exposure altered fecal metabolome of mice

**Figure 6:**
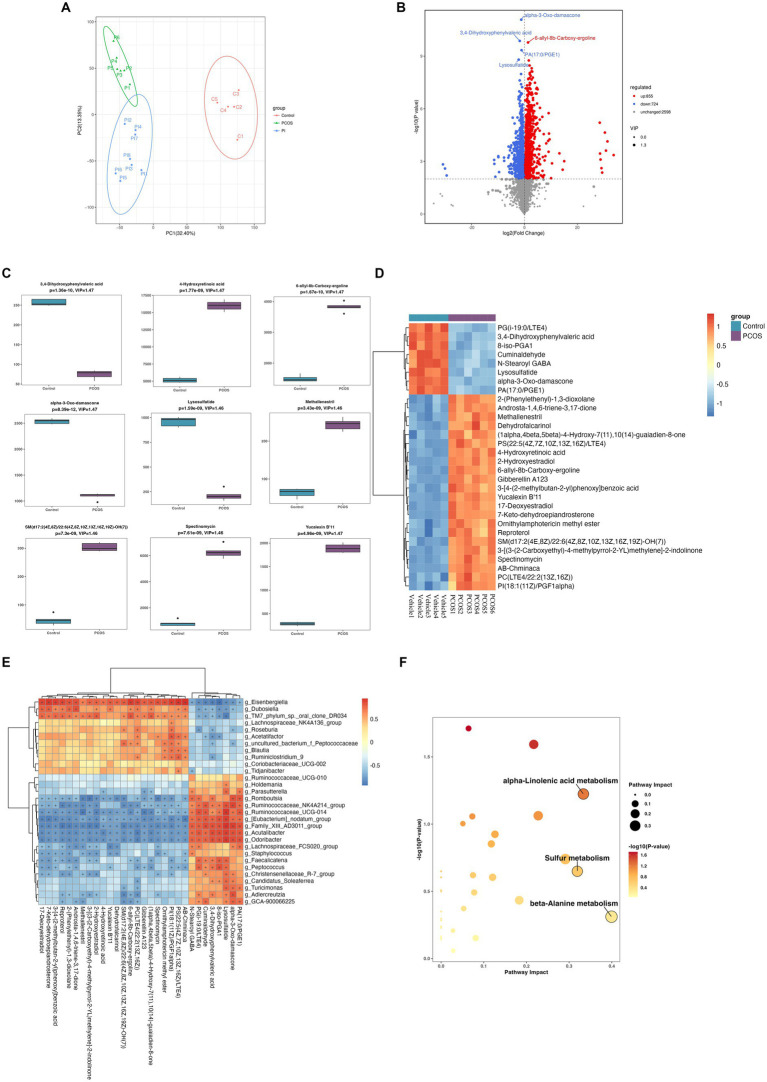
Non-target metabolomic analysis was performed in vehicle and PCOS mice. **(A)** Principal component analysis of fecal metabolites from three groups of mice, **(B)** Volcano plot showing differential expression of metabolites, **(C)** Boxplot displaying the abundance of the top nine differential metabolites, **(D)** Heat map presenting the abundance of the top 30 differential metabolites detected in fecal samples, **(D)** The correlation analysis between the differential metabolites and gut microbiota at the genus level. **(F)** Bubble plot shows pathway of differentially enriched metabolic pathways. Each bubble indicates an enriched pathway. Horizontal axis and size of the bubble indicate the impact of the pathway. Vertical axis and color of the bubble indicate the significance of enrichment. *p < 0.05, **p < 0.01, ***p < 0.001, and vehicle: *n* = 5, PCOS: *n* = 6 and PCOS +irisin: *n* = 8.

The fecal untargeted metabolomics was carried out to further understand the alterations of the gut chemical environment in PCOS mice and PCOS mice treated with r-irisin. Principal component analysis (PCA) of the metabolite profiling from the three groups of mice is shown in Figure 6A. By differential analysis of metabolites according to the differential generation criteria in methods, 1,579 differential metabolites were obtained from the PCOS group and the vehicle group (control), of which 855 increased and 724 decreased (see volcano plot of Figure 6B). We then plotted the top 30 and the top 9 differential metabolites with the largest -log10 *p*-value in abundance heatmap and boxplots, respectively. Among these 22 significantly increased metabolites, there were 2-(Phenylethenyl)-1,3-dioxolane, Androsta-1,4,6-triene-3,17-dione, and methallenestril, and among the eight significantly decreased metabolites, there were PG(i-19:0/LTE4), 3,4-dihydroxyphenylvaleric acid, and 8-iso-PGA1 (Figures 6C,D). Furthermore, the correlation analysis between differential metabolites and gut microbiota showed that the relative abundance of metabolites AB.Chminaca, Lysosulfatide, and X7.Keto.dehydroepiandrosterone were positively correlated with the relative abundance of the genus *Eisenbergiella* and *Family _ XIII _ AD3011 _ group* (Figure 6E). Meanwhile, the pathway enrichment analysis of differential metabolites showed that several key metabolic pathways including α-linolenic acid metabolism, sulfur metabolism, and β-alanine metabolism were affected by the exposure to DHEA + HFD (Figure 6F).

### 3.7 R-irisin treatment altered fecal metabolome in PCOS mice

**Figure 7:**
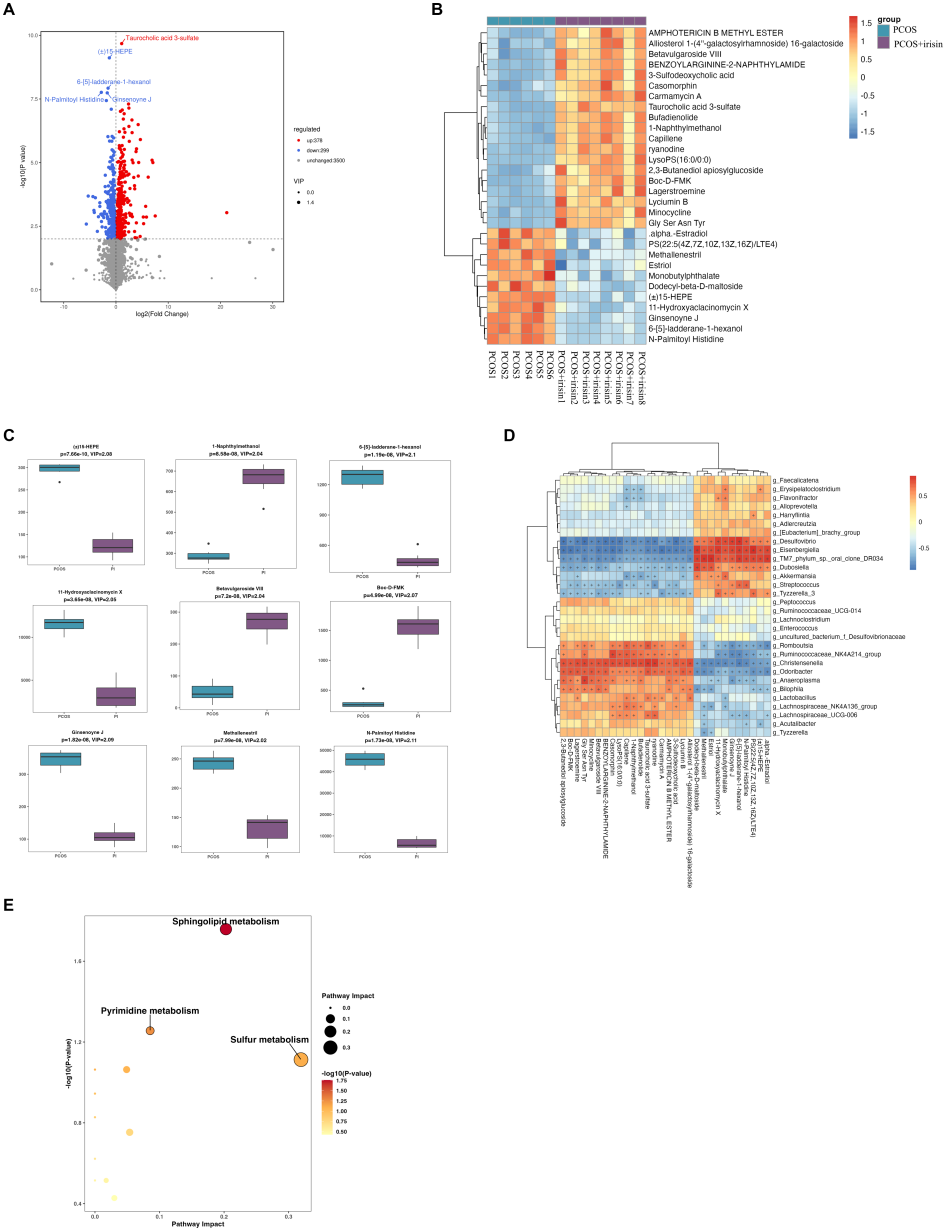
Non-target metabolomic analysis was performed in PCOS and PCOS +irisin mice. **(A)** Volcano plot showing differential expression of metabolites; **(B)** Boxplot displaying the abundance of the top nine differential metabolites; **(C)** Heat map presenting the abundance of the top 30 differential metabolites detected in fecal samples. **(D)** The correlation analysis between the differential metabolites and gut microbiota at the genus level. **(E)** Bubble plot shows pathway of differentially enriched metabolic pathways. Each bubble indicates an enriched pathway. Horizontal axis and size of the bubble indicate the impact of the pathway. Vertical axis and color of the bubble indicate the significance of enrichment. *p < 0.05; **p < 0.01; and ***p < 0.001, vehicle: *n* = 5; PCOS: *n* = 6 and PCOS +irisin: *n* = 8.

Through non-targeted metabolomics studies, we found 667 differential metabolites in the feces between PCOS and PCOS + irisin mice, including 378 increased metabolites and 299 decreased metabolites (Figure 7A). Similarly, we extracted the top 30 and top 9 differential metabolites with the smallest p-value and plotted into the abundance heat map and boxplot, respectively, and found that after the r-irisin treatment, methallenestril and PS (22:5 (4Z, 7Z, 10Z, 13Z, 16Z) / LTE4) in the fecal metabolites were significantly lower compared with the PCOS group, suggesting that r-irisin can reverse the levels of these two metabolites toward healthy control mice (Figures 7B,C). Moreover, the correlation analysis showed that the two different metabolites were consistent with the changes in the genus level *Eisenbergiella* and *Dubosiellad* (positive correlation, Figure 7D). Additionally, pathway enrichment analysis of differential metabolites illustrated r-irisin-altered pathways including sphingolipid metabolism, pyrimidine metabolism, and sulfur metabolism (Figure 7E).

## 4 Discussion

Hyperandrogenism and insulin resistance (IR) are common phenotypes of PCOS. Admittedly, numerous studies show the relationship between gut microbiota and a variety of chronic conditions. However, the relationship between gut microbiota, insulin resistance, and hyperandrogenism was intricate in PCOS. Excessive insulin causes hyperandrogenism. That is, on the one hand, superfluous insulin produces excessive luteinizing hormone (LH), producing excessive androgen from the ovary and the adrenal gland. On the other hand, the synthesis of sex-binding globulin (SHBG) was inhibited (He and Li, 2020). Conversely, hyperandrogenism damages hepatic and peripheral insulin sensitivity (Nader, 1991). In addition, gut microbiota may change under various sex hormones. For instance, evidence shows that no significant difference was found between men and women in the pre-puberty period. Then, during the adolescent period, with the increased androgens, alpha diversity decreased more in men than in women, which is consistent with rats. After ovariectomy in rats, this difference was disappeared (Moreno-Indias et al., 2016). These studies revealed that serum sex hormones might affect gut microbiota to some extent. Conversely, gut microbiota takes part in androgen metabolism. To be specific, beta-glucuronidase in some gut microbiota unconjugates the glucuronidated androgen and elevated the free androgen. In addition, bacteria such as *Butyricicoccus desmolans* and *Clostridium cadaveris* express steroid-metabolizing enzymes (Colldén et al., 2019; Li et al., 2022). Individuals with IR also show differences in gut microbiota, and fecal microbiota transplantation (FMT) changes insulin sensitivity. Insulin resistance appeared in germ-free mice after transplanting intestinal flora of healthy mice. Similarly, insulin sensitivity ameliorated in recipients after transplanting the gut microbiome of healthy people (He and Li, 2020). These studies showed the interaction between gut microbiota, insulin resistance, and hyperandrogenism. The change in gut microbiota might be the result and the cause of the PCOS phenotype. Moreover, amelioration of gut microbiota might improve the PCOS phenotype (Starling, 2021; Salehi et al., 2024; Sun et al., 2024).

Through this study, from the perspective of gut microbiota and fecal metabolomics, we investigated for the first time the role and mechanism of the exercise sensor molecule irisin on the reproductive endocrinal-metabolic phenotype in PCOS model mice. Here, we found that the r-irisin treatment could alleviate reproductive endocrinal abnormalities of the PCOS mice, including the decrease in androgen levels, restoration of ovarian morphology, increase of follicle developmental regulatory gene expression, and inhibition of the expression of apoptosis gene. Through the detection and analysis of mouse fecal microbiome and metabolite, we found that there were significant and extensive differences in fecal microbial abundance and metabolite levels between PCOS mice and healthy control mice, and there were also significant differences in fecal flora and metabolite between PCOS and PCOS mice treated with r-irisin, indicating that irisin may affect the phenotype of PCOS by intestinal microbes and metabolites.

Currently, irisin is considered to be one of the mediator molecules involved in physical activity. It is mainly synthesized during the muscle movement and secreted into the bloodstream. In endocrine function, it acts on the target organs or cells, thereby affecting their biological function, such as the conversion of white adipose tissue (WAT) to brown adipose tissue (BAT), and regulating oral hard and soft tissues and vascular calcification (Pedersen and Febbraio, 2012; Saleem and Ghafoor, 2023; Grzeszczuk et al., 2024; Wang et al., 2024). Recently, irisin was found to enhance the intestinal barrier in NOD mice, postponing the onset of type 1 diabetes (Sun et al., 2024). Our published study found that deletion of FNDC5, the precursor of irisin, and reduction in fertility in female adult mice led to abnormal glucose and lipid metabolism, indicating that irisin has regulatory effects on both metabolism and reproductive function (Luo et al., 2020). This led us to speculate that, when using r-irisin as a therapeutic agent, it may directly improve the symptoms of PCOS, which is a typical chronic disease with glucose-lipid metabolism dysfunction and reproductive endocrine dysfunction. On the other hand, an important factor in the development of PCOS is high androgen; while in this study, we found that androgens are the most sensitive sex hormone after r-irisin treatment of PCOS mice, which is similar to the results of a previous experimental study on PCOS rats by Zheng et al. (2022). However, there is a reciprocal relationship between androgen levels and obesity and insulin resistance, and it is unclear whether the androgen-lowering effect of irisin in PCOS mice is indirect or direct. In our study, irisin did not improve insulin sensitivity and glucose metabolism in PCOS mice. In other studies, it is not consistent whether irisin alleviates insulin resistance. Previous studies showed that mice were prone to insulin resistance by globally deleted irisin via CRISPR/Cas-9 and high-fat-diet (HFD) (Wang et al., 2023). In addition, the insulin resistance of HFD-treated bilateral ovariectomy rats was reduced by irisin therapy (Gheit et al., 2022). However, chronic voluntary wheel running exercise ameliorates metabolic index in HFD-induced obese C57BL6J mice, while no difference was found in serum irisin (Cho et al., 2023). We speculate that it is probable that the strains of the experimental animals are the cause of the discrepancy. Mice seem more likely to exhibit insulin resistant than rats (McNeilly and Duncan, 2013), and C57BL/6 strain is more pronounced in the diabetes-prone strain than in the BALB/cByJ strain (Dowling et al., 2013). It might be that the strain *per se* neutralizes the beneficial effect of irisin on metabolic parameters. In addition, drugs alleviate PCOS by central modulation (hypothalamic–pituitary–gonadal axis) or peripheral target (insulin resistance, gut microbiota, immune inflammatory cytokines, or others) (Liao et al., 2021; Liu et al., 2021). Here, irisin may decrease the testosterone directly or by alleviating gut microbiota without a change in insulin.

In the study of gut microbiome, we found that genera *Eisenbergiella* and *Dubosiella* were significantly increased in feces of the PCOS model group compared with control mice, which was significantly decreased after r-irisin intervention. It is interesting that *Dubosiella* increased in the PCOS group in our study. There was only one study now elaborated on the abundance of *Dubosiella* in PCOS. It was found that water extract of *A. sinensis* root, a traditional Chinese medicine, could increase the abundance of *Dubosiella.* They hold the view that *Dubosiella* is a probiotic (Gao et al., 2023). However, another study showed that more *Dubosiella* were found in male mice than in female mice at 8 weeks, which were lesser in male mice at 6 weeks (Ortiz-Alvarez de la Campa et al., 2024). It revealed that the elevated abundance of *Dubosiella* might be caused by the raised testosterone. Moreover, in our study, irisin might ameliorate the hyperandrogenism in PCOS, thereby decreasing *Dubosiella* in PCOS mice. More studies need to be conducted to verify this speculation. These two bacteria may play a role in regulating host energy metabolism and maintaining the intestinal environment and host immune homeostasis (He et al., 2020; Martin-Gallausiaux et al., 2021). They may also be associated with some unhealthy conditions, such as hemorrhagic stroke, schizophrenia, and attention deficit (Jakobi et al., 2023; Shen et al., 2023; Misiak et al., 2024). Thus, they may not be “probiotics” in any situation. The pros and cons of these two bacteria are still unknown. In addition, we also detected that the abundance of *Odoribacter* decreased in PCOS mice and increased after irisin intervention. *Odoribacter* is considered a short-chain fatty acid (SCFA)-producing beneficial bacterium. Studies have found that it can improve glucose metabolism and inflammatory response in diabetic model mice, and its reduced abundance is also significantly associated with enhanced inflammatory response, obesity, diabetes, elevated blood pressure, and other diseases (Liu et al., 2017; Angoorani et al., 2021; Huber-Ruano et al., 2022; Vallianou et al., 2023). In addition, we can observe that, at the genus level, *Lachnospiraceae nk4a136* bacteria significantly increased after r-irisin treatment, becoming one of the signature bacteria of the r-irisin treatment group, and this bacterium is also a SCFA-producing bacterium, which is decreased in the condition of obesity and IR and increased in the condition of a healthy lifestyle or benign intervention such as spermidine and resveratrol (Ma et al., 2020; Wang et al., 2020; Muralidharan et al., 2021). These results indicate that r-irisin treatment may have both beneficial and negative effects on the gut microbiota, and the beneficial effects of the drug on improving PCOS phenotypes may be, at least in part, due to changes in the gut microbiota.

In the study of untargeted fecal metabolomics, we found that Methallenestril and PS (22:5 (4Z, 7Z, 10Z, 13Z, 16Z) / LTE4) were found to be significantly increased in the PCOS model group and significantly decreased after r-irisin intervention, suggesting that these two metabolites may be important markers of r-irisin affecting the PCOS phenotype. Methallenestril is an estrogen analogue with mild estrogen activity and possibly has an effect on ovarian function (Goldfarb and Napp, 1956; Schneeberg et al., 1956). However, the specific correlation of this metabolite with PCOS needs further study. The other metabolomics, PS (22:5 (4Z, 7Z, 10Z, 13Z, 16Z) / LTE4), is an oxidized phosphatidylserine (PS). Similar to all oxidized lipids, oxidized phosphatidylserine is considered a class of biomolecules transducing cell signaling and regulating cell metabolism (Hajeyah et al., 2020). However, the significance of the changes in the fecal metabolites in this study needs to be explored through further studies.

Our study provides clues for the treatment of irisin on PCOS to some extent. Irisin might improve the PCOS phenotype by targeting the gut microbiota. However, more studies should be conducted because the direct molecular targets are still unclear. Generally, the clinical usage of new drugs is time-consuming and labor-intensive, which are derived from combinations of application of biomedical engineering and computer application technology, such as drug-target interactions (DTIs), drug-disease associations (DDAs), and micro-disease associations. These new technologies bring new approaches to disease-mechanism research, translational medicine, and drug repurposing, saving time and expenditure (Pushpakom et al., 2019; Gao et al., 2023; Zhao et al., 2023). From this perspective, our study collected raw data on the effects of irisin on PCOS in microbiology, providing part of the basis for PCOS mechanism and application of irisin. We believe that there will be a virtuous circle between the increasing raw data and the association and application of irisin-microbiome-PCOS.

## 5 Conclusion

Although PCOS is a serious health problem worldwide, limited treatment options focus primarily on symptoms, producing unsatisfactory treatment effects and long-term metabolic complications, mainly because of a lack of understanding of the etiology. Intestinal dysbiosis is a characteristic of PCOS patients and rodent models. Therefore, manipulation of the gut microbiota by fecal microbiota transplantation (FMT), probiotics, prebiotic, or commensal bacteria improved the PCOS phenotype (Qi et al., 2019, 2021; Han et al., 2021; Rodriguez Paris et al., 2022). Though there are many variables influencing the traits of intestinal flora, it can be stated that a significant share of these variables is related to diet and activity (Sivasankari and Usha, 2022). However, as a molecule of exercise sensor, there was only one published article regarding the influence of irisin on gut microbiome and metabolomics in ulcerative colitis animal models (Huangfu et al., 2021). In this study, we demonstrated, once again in the PCOS mouse model, that irisin treatment alone can change the intestinal flora and metabolite composition, suggesting that intestinal flora is the target of irisin regulation, and irisin may at least partially simulate the role of exercise in the treatment of PCOS.

## Data availability statement

The datasets presented in this article are not readily available because of data access limitations imposed by Biomarker Technologies Corporation Co., Ltd, who carried out the untargeted metabolomic analysis. Requests to access the datasets should be directed to the corresponding author.

## Ethics statement

The animal study was approved by Medical Ethics Committee of West China Second University Hospital, Sichuan University (Approval No. WCSUH21-2019-089). The study was conducted in accordance with the local legislation and institutional requirements.

## Author contributions

MY: Data curation, Formal analysis, Investigation, Methodology, Writing – original draft, Writing – review & editing. HD: Software, Visualization, Writing – review & editing. SZ: Data curation, Formal analysis, Investigation, Methodology, Writing – review & editing. DL: Data curation, Formal analysis, Investigation, Methodology, Writing – review & editing. XS: Supervision, Writing – review & editing. LH: Supervision, Writing – review & editing. YC: Data curation, Formal analysis, Methodology, Project administration, Software, Visualization, Writing – original draft, Writing – review & editing. LX: Project administration, Resources, Supervision, Writing – review & editing.
