# Supplementary material for: Irisin alleviated the reproductive endocrinal disorders of PCOS mice accompanied by changes in gut microbiota and metabolomic characteristics
Source: Front Microbiol. 2024 May 23;15:1373077. doi: 10.3389/fmicb.2024.1373077 (PMC11153696; doi:10.3389/fmicb.2024.1373077)
Supplement: Supplementary file 1 [file Table_1.doc]

Supplement Table 1. Instruments used in the experiment.

| Instruments | Manufacturer | Model |
| --- | --- | --- |
| Blood glucose meter | AUCC | Guide Me |
| Microscope | Wanneng | 80I |
| Precise analytical balance | Liangping | JA5003 |
| Centrifuge | Eppendorf | 5804, 5425R |
| Radio-Immunity Analyzer | Xian Nuclear Instrument Factory | XH6080 |
| Gradient gene amplifier | Appliedbiosystems | veriti96well 9902 |
| Ultra Performance Liquid Chromatography，UPLC | Waters | Acquity I-Class PLUS |
| High-Resolution Mass Spectrometry,HRMS | Waters | Xevo G2-XS QTOF |
| Chromatographic column | Waters | HSS T3 column (1.8um 2.1*100mm) |
